# Quantitative dissection of variations in root growth rate: a matter of cell proliferation or of cell expansion?

**DOI:** 10.1093/jxb/ery272

**Published:** 2018-07-25

**Authors:** Chvan Youssef, François Bizet, Renaud Bastien, David Legland, Marie-Béatrice Bogeat-Triboulot, Irène Hummel

**Affiliations:** 1 Université de Lorraine, AgroParisTech, INRA, UMR Silva, Nancy, France; 2 UMR PIAF, INRA, Université Clermont Auvergne, Aubière, France; 3 Department of Collective Behaviour, Max Planck Institute for Ornithology, University of Konstanz, Konstanz, Germany; 4 UMR Biopolymers, Interactions and Assemblies, INRA, Nantes, France

**Keywords:** Cell expansion, cell division, kinematics, elemental elongation rate, *Populus*, root apical meristem, root growth rate

## Abstract

Plant organ growth results from cell production and cell expansion. Deciphering the contribution of each of these processes to growth rate is an important issue in developmental biology. Here, we investigated the cellular processes governing root elongation rate, considering two sources of variation: genotype and disturbance by chemicals (NaCl, polyethylene glycol, H_2_O_2_, abscisic acid). Exploiting the adventitious rooting capacity of the *Populus* genus, and using time-lapse imaging under infrared-light, particle image velocimetry, histological analysis, and kinematics, we quantified the cellular processes involved in root growth variation, and analysed the covariation patterns between growth parameters. The rate of cell production by the root apical meristem and the number of dividing cells were estimated *in vivo* without destructive measurement. We found that the rate of cell division contributed more to the variation in cell production rate than the number of dividing cells. Regardless of the source of variation, the length of the elongation zone was the best proxy for growth rate, summarizing rates of cell production and cell elongation into a single parameter. Our results demonstrate that cell production rate is the main driver of growth rate, whereas elemental elongation rate is a key driver of short-term growth adjustments.

## Teaser

Distinct cellular processes trigger root growth regulation: cell elongation rate drives rapid growth response to environment while cell division rate determines the growth capacity of individual roots.

## Introduction

Plant organ growth results from the combination of two processes: cell production and cell expansion. Understanding how cell division and cell elongation, and their balance, contribute to root elongation rate is an important issue in developmental biology (Gázquez and Beemster, 2017; Yang *et al.*, 2017). In organs that grow linearly such as roots, growth rate is integral to the elemental elongation rate (EER) along the growth zone (Silk, 1992). In the root apical meristem (RAM), cell elongation is low, accounting for less than 15% of the total root growth, and most growth is due to rapid elongation occurring in the elongation zone (Bizet *et al.*, 2015). Cell division rate also impacts on the root elongation rate as the cells produced by the RAM feed the elongation zone (Baskin, 2013). In previous studies, the dynamics of cellular processes have been studied either by quantifying their local distribution within the growth zone (Pahlavanian and Silk, 1988; Sharp *et al.*, 1988; Hukin *et al.*, 2002) or by calculating integrative growth parameters (e.g. cell production rate by the RAM, number of dividing cells in the RAM, residence time in the elongation zone) (Baskin, 2000; Beemster *et al.*, 2002; Fiorani and Beemster, 2006). For instance, the local pattern of cell division rate along the RAM has been computed from spatial profiles of velocity and cell length (Sacks *et al.*, 1997). By contrast, under steady-state conditions (or assuming the time-invariance of growth processes) cell production rate has been calculated as the root elongation rate divided by the length of mature cells (Beemster *et al.*, 2002). Similarly, assuming that all cells within the RAM are dividing (Ivanov *et al.*, 2002), the average cell division rate can be estimated from the ratio of the cell production rate to the number of dividing cells (Beemster and Baskin, 1998).

A better understanding of the mechanisms driving variation in root growth has been obtained by kinematics—a powerful mathematical framework with which to analyse the spatial distribution of growth (Silk and Erickson, 1979; Sharp *et al.*, 1988, 2004; Beemster *et al.*, 2002; Walter *et al.*, 2009; Royer *et al.*, 2016). For instance, the developmental acceleration of root growth has been shown to be accompanied by increased cell production with little change in cell expansion rate (Beemster and Baskin, 1998). In contrast, cell production rate together with cell elongation were found to be responsible for the genetic differences observed in root elongation rate between genotypes of Arabidopsis and across plant species (Beemster *et al.*, 2002; Gázquez and Beemster, 2017). Kinematics has also been used to dissect and model the interplay of hormones in the control of root growth (Beemster and Baskin, 2000; Rahman *et al.*, 2007; Band *et al.*, 2012; De Vos *et al.*, 2014). Physiological examination has revealed, for instance, that ethylene inhibits root growth by targeting the EER and reducing the length of the elongation zone (Swarup *et al.*, 2007). A similar response was seen in the presence of cytokinin (Beemster and Baskin, 2000). Finally, root growth is known to be highly responsive to environmental cues, and interferences under stress have been widely documented under the spotlight of kinematics (Baskin, 2013).

Nowadays, many tools are available for efficient kinematic analysis (van der Weele *et al.*, 2003; Basu *et al.*, 2007; Chavarría-Krauser *et al.*, 2008; Wuyts *et al.*, 2011; Iwamoto *et al.*, 2013; Bastien *et al.*, 2016; Yang *et al.*, 2017). Various automatic tracking algorithms have enhanced particle image velocimetry techniques, providing high-resolution growth profiles compatible with different kinds of imaging technology. In particular, the use of natural marks on the root has heralded significant progress for non-invasive kinematics. Infrared illumination has been shown to be a simple way to enhance the natural texture of the root efficiently in various species and in organs of different sizes (Walter *et al.*, 2002; Bizet *et al.*, 2015; Bastien *et al.*, 2016). Recent work in our laboratory has shown that RAM length can be monitored *in vivo* from infrared reflectance profiles (Bizet *et al.*, 2015, 2016).

In this context, we investigated the relative contribution of cell division and cell elongation to the variation in root elongation rate in *Populus*. Due to its economic interest, high genetic diversity, and numerous molecular resources, *Populus* has reached the status of a model tree (Plomion *et al.*, 2016). Poplars are among the fastest growing trees and their high adventitious rooting capacities make this genus an ideal system for examining root growth. We first considered the interspecific variability in root growth, and quantified growth rate and underlying cellular processes for eight poplar genotypes. We then addressed the question of the contribution of cellular processes to growth variation induced by external cues. For that purpose, the root growth of one poplar genotype was quantified in the presence of various chemical treatments selected for their known impact on growth. These two experiments were designed to increase the range of root elongation rates under scrutiny, allowing a thorough analysis of covariation patterns. The same rationale was followed for the analysis of diversity and of chemically induced growth disturbances. Analysis of covariation patterns under the two sources of variation revealed the cellular processes that determine growth rate under optimal growth conditions and during the adjustment to disturbance by chemicals.

## Materials and methods

### Plant material, growth conditions, and treatments

Homogeneous woody cuttings of eight poplar genotypes belonging to different species or commercial hybrids (*Populus alba* L. cv. Villa franca, *P. nigra* cv. 6J29 and N38, and *P. deltoides*×*P. nigra* cv. Carpaccio, Flevo, I214, Lambro, and Soligo) were grown in hydroponics in a half-strength Hoagland nutrient solution as described previously (Bizet *et al.*, 2015). Cuttings emitted between 1–8 adventitious roots after ~10 d in a dark room (air temperature 23 ± 1 °C; atmospheric humidity 45 ± 11%). Once a root reached 2 cm long, the cutting was transferred to a transparent tank filled with aerated and circulating nutrient solution (Bizet *et al.*, 2015). Only one root per cutting was selected for root growth monitoring, chosen only on the basis of its length. To ensure the quality of imaging, the dehiscent part of the root cap was carefully removed (at least 1 h before imaging) so that root growth was only slightly and transiently disturbed. Growth analysis was performed once growth had recovered. No other manipulation of the root occurred until the end of the experiment.

The first experiment (Exp1) was designed to characterize root growth variability across the eight poplar genotypes, and was carried out from July to November 2016, using a random design for replication per genotype (*n*=7–13 roots). The second experiment (Exp2) examined the response of the Flevo genotype to different treatments, and was carried out from February to June 2017, using a random design for replication per treatment (*n*=3–12 roots). In Exp2, treatments were applied by adding chemicals to the nutrient solution without manipulation of the roots (Royer *et al.*, 2016; Bizet *et al.*, 2017). Roots were subjected to either 70 mM NaCl or 160 g l^−1^ polyethylene glycol (PEG 4000, Merck Chemicals, Darmstadt, Germany), generating osmotic potentials of –0.30 MPa and –0.37 MPa, respectively (measured with a Wescor 5500; Logan, UT, USA). Roots were also subjected to either 2 µM or 10 µM of ABA (Sigma-Aldrich, St. Louis, USA), or 2 mM H_2_O_2_ (30% w/w, Sigma-Aldrich). The oxygen level compared to controls was not affected by any of these treatments (measured with a HQ40D oximeter; Hach Lange, Noisy-le-grand, France; data not shown).

### Time-lapse imaging, kinematic and infrared picture analyses

In both experiments, root growth was monitored under infrared light as described previously (Bizet *et al.*, 2017). Infrared light does not stimulate photoreceptor systems, nor does it affect root growth or generate tropism (Kiss *et al.*, 2003; Wiese *et al.*, 2007; Ivakov *et al.*, 2017). Images were taken every 6 min for 1 h and the camera was computer-controlled with digiCamControl (V2.0.0; http://digicamcontrol.com). In Exp1, images were taken at a resolution of 1.6 µm pixel^−1^ (camera Nikon 5200, macro lens Nikkor 60 mm, with a 56-mm extension tube). In Exp2, images were taken at a resolution of 2.6 µm pixel^−1^ (same settings except a 20 mm extension tube was used). In Exp2, time-lapse imaging was performed before treatment onset, as well as 1 h, 2 h and 24 h after treatment onset.

For each root, velocity and EER profiles were obtained by kinematics using the dedicated software KymoRod v0.11.0 (Bastien *et al.*, 2016). For a stack of time-lapse images, KymoRod computes the skeleton of the root contour, automatically extracts its midline, and calculates the curvilinear abscissa along the midline. Here, image smoothing was achieved by a box filter radius of 2 and the root contour was computed from a threshold value set individually for each root. Median elongation was then calculated using Rod-PIV (Bastien *et al.*, 2016), with spatial smoothing Lx=0.3. By default, a time-step of 6 min between images was used (providing nine profiles of velocity over 1-h-long time-lapse imaging); however, the time-step was set to 12 min when the growth rate was low (to ensure that displacement was large enough for reliable particle image velocimetry, PIV). The correlation window was set at 50 pixels to remain within the root boundaries. The smoothing window for calculation of EER took 50 points.

The velocity and EER profiles, as well as the skeleton of the first image, were extracted from the KymoRod file with R (v3.3.1, http://www. R-project.org, using the R.matlab package). The skeleton midline was projected onto the respective raw image using Fiji (Schindelin *et al.*, 2012), brightness profile along the midline was measured after thickening the skeleton midline to the root borders and smoothing the image (Gaussian blur filter, radius 20, as previously described by Bizet *et al.*, 2015). The position of the quiescent center (QC) was determined visually on the image and confirmed by a depression in the brightness profile. Since the origin in KymoRod data is the apical point of the root skeleton (including the root cap), the data were translated so that the QC was the origin of the profile and the velocity was zero at the QC. The profiles of velocity and EER were inspected visually to remove unreliable data due to artefactual imaging. The shootward border of the RAM was determined from the relative brightness profile with a threshold of 75%, as in Bizet *et al.* (2015). The root diameter within the growth zone (Diam, mm) was recorded on the same image.

### Histological analysis

At the end of growth monitoring, the root apices were fixed in 4% paraformaldehyde in a phosphate-buffered saline solution for 30 min under vacuum (*n*=4–5 roots per genotype or treatment). Fixed samples were rinsed three times with distilled water then dehydrated in a graded ethanol series and embedded in Technovit 7100 resin (Heraeus Külzer embedding kits) following the manufacturer’s instructions. Samples were stored at 35 °C for at least 4 d. Longitudinal sections of 5-μm thick were cut in the middle of the root with a rotary microtome (Microm HM355S, Thermo Scientific). The sections were stained with Toluidine Blue O, mounted in synthetic resin (Eukitt^®^) and examined under a DMLB Leica microscope equipped with a Leica DFC420C camera (Leica Microsystems). Longitudinal sections were imaged at 100× magnification. Images were assembled using MosaicJ (Thévenaz and Unser, 2007). Cortical cell length and its distance from the QC were measured semi-automatically from longitudinal sections using a macro in Fiji (Bizet *et al.*, 2015). The cell length profile along the apex was interpolated with a cubic smoothing spline using R (smooth.spline, spar=0.9 to 1).

### Determination of growth parameters

Growth parameters were extracted automatically from the EER and velocity profiles using a script in R and their values averaged for a given time-point. Overall root elongation rate (ORER, mm h^−1^) was determined as the maximal velocity. EER_max_ (h^−1^) was the maximum of EER. The shootward border of the growth zone was set at the position where EER dropped below 3%. The length of the elongation zone (EZ, mm) was determined as the distance between the shootward borders of the growth zone and the RAM.

For about half of the roots in both experiments (*n*=50 roots), the cell flux profile was calculated by the ratio of velocity (using the last velocity profile) to cell length (using the smoothed profile of cell length), and the local rate of cell production (CPR, cells mm^−1^ h^−1^) was calculated as the spatial derivative of the cell flux (using the Erickson five-point formula; Erickson, 1976). The rate of cell production by the RAM (*P*, cells h^−1^) corresponded to the maximal cell flux. In steady state, *P* is classically determined as the ratio between the ORER and the mature cell length. Since cell flux is constant and maximal from the RAM shootward border to the mature zone, P was determined at the RAM shootward border as the ratio between velocity and the mean cell length at the RAM shootward border, which was defined as the position where CPR equalled zero. A proxy for *P* was computed as the ratio between velocity at the RAM shootward border determined by the brightness profile (Bizet *et al.*, 2015) and 21.5 µm. Biologically, this scaling factor corresponds to the maximal cell length observed within the cell proliferation domain of the RAM (Bizet *et al.*, 2015). The number of dividing cells (*N*_div_) can be expressed as the ratio between the RAM length and the average cell length within the RAM. *N*_div_ was therefore calculated from the RAM length determined at CPR=0 and the mean of all cell lengths measured in the RAM. A proxy for *N*_div_ was computed from the RAM length determined by the brightness profile divided by 14 µm. Biologically, this scaling factor corresponds to the average cell length in the cell proliferation domain of the RAM (Bizet *et al.*, 2015). The average cell division rate (*D*, h^−1^) was defined as the ratio between the cell production rate *P* and *N*_div_.

**Fig. 1.**
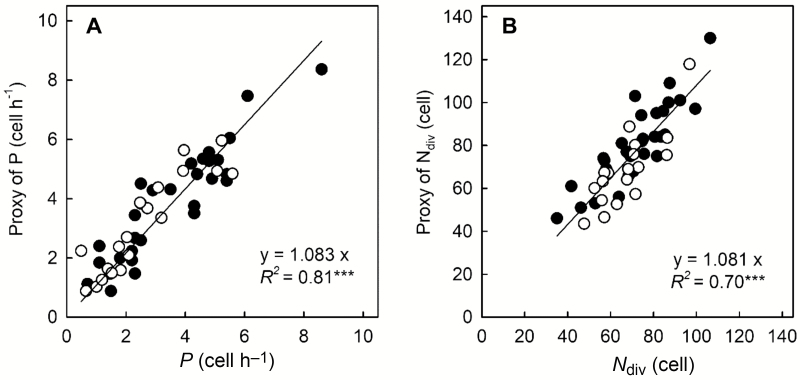
*In vivo* determination of cell production rate and of number of dividing cells. (A) Relationship between cell production rate (*P*), calculated as the ratio between velocity and cell length at the shootward border of the RAM, and its proxy. (B) Relationship between number of dividing cells (*N*_div_), calculated as the ratio between RAM length and the mean length of dividing cells, and its proxy. Closed circles indicate roots from Exp1 and open circles roots from Exp2. The linear regressions are plotted, and the significance of the correlations is indicated (***P≤0.001).

For both *P* and *N*_div_, the relationships between the proxy and its respective reference (determined at the position where CPR equals zero) covered a large range of values, were linear, and robust over genotypes and treatments (*R*^2^=0.84, *n*=50, *P*<0.001; *R*^2^=0.72, *n*=50, *P*<0.001, respectively, Fig. 1A, B). Figure 1 highlights that *P* and *N*_div_ were estimated properly using a non-invasive method based on the brightness profile generated by infrared illumination (providing pertinent scaling factors of cell length), which requires very low experimental effort as compared to histological methods. *In vivo* proxies of *P* and *N*_div_ were computed for the full set of roots and were used in covariation analyses. Statistical and principal component analyses (PCA) were performed with R (ade4 package).

## Results

### Variability in growth parameters across poplar genotypes

**Fig. 2.**
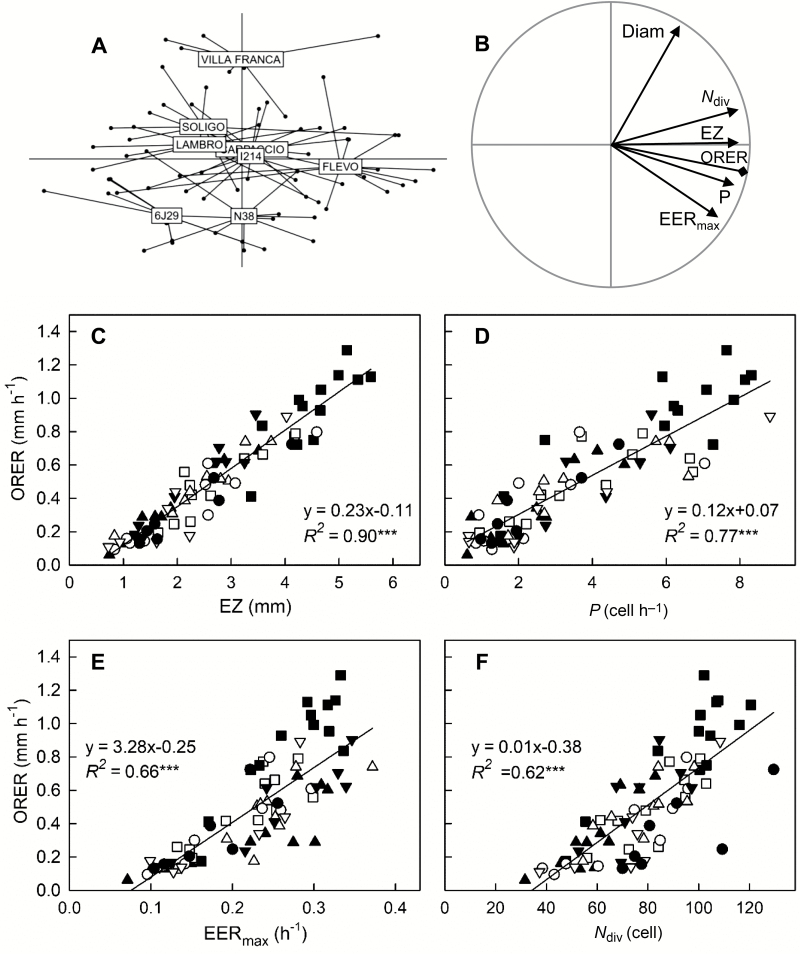
Covariation of growth parameters across genotypes. (A, B) Principal component analysis (PCA) was performed on the growth parameters of 75 roots from eight *Populus* genotypes (Exp1). The first two main factors of the PCA account for 88% of the total inertia. For clarity, individuals and variables are displayed on the same PCA plane on distinct figures. (A) Projection of the individuals in the F1×F2 plane. Each point is a root. Lines and boxes indicate genotype barycentres. (B) Projection of growth parameters in the F1×F2 plane. *P*, cell production rate; *N*_div_, number of dividing cells; EZ, length of the elongation zone; EER_max_, maximal elemental elongation rate; and Diam, the apical root diameter. Overall root elongation rate (ORER) was added as a supplementary variable. (C–F) Pairwise relationships between ORER and growth parameters. The *Populus* genotypes are indicated as follows: closed triangles, 6J29; closed inverted triangles, N38; open squares, Carpaccio; closed squares, Flevo; open triangles, I214; open circles, Lambro; open inverted triangles, Soligo; and closed circles, Villa Franca. Linear regressions are plotted and the significance of the correlations is indicated (***P≤0.001).

In Exp1, eight poplar genotypes were grown in optimal nutrient solution and root growth was monitored independently for 75 roots. A 21-fold variation in overall root elongation rate (ORER) was found, ranging from 0.06 mm h^−1^ for a root of 6J29 and to 1.29 mm h^−1^ for a root of Flevo. To assess the contribution of the growth parameters to ORER variability, a principal component analysis was performed on the matrix of growth parameters and root diameter, using ORER as a supplementary variable (Fig. 2A, B). Root diameter was included to take into account the diversity in root morphology. The first and second principal components (PCs) explained 66% and 22% of the total variance, respectively, with PC3 accounting for less than 5%. PC1 was highly and positively related to the length of the elongation zone (EZ), to the number of dividing cells (*N*_div_), and to the cell production rate by the RAM (*P*). The maximum elemental elongation rate (EER_max_) also contributed positively to PC1 but to a lesser extent. As shown by the projection of ORER, PC1 discriminated fast-growing roots from slow-growing ones, with a large inter-individual dispersion around the barycentre of the genotypes (Fig. 2A, B). Flevo roots exhibited a significantly longer elongation zone (EZ=4.3 ± 0.3 mm; mean ±SE) and faster growth rate (ORER=0.88 ± 0.09 mm h^−1^) than all other genotypes (EZ=2.6 ± 0.1 mm, *P*<0.001; ORER=0.5 ± 0.1 mm h^−1^, *P*<0.001). Focusing on the barycentre of genotype ellipses revealed PC2 as a key structuring factor for genotype diversity. PC2 separated *P. nigra* (6J29 and N38) from commercial hybrids and *P. alba* (Villa Franca), mostly through the difference in root apex diameter (Diam). Villa Franca roots were significantly thicker (Diam=0.82 ± 0.03 mm) as compared to other genotypes (Diam=0.57 mm±0.02; *P*<0.001). A weak but significant correlation was found between ORER and Diam when considering all the genotypes (*R*^2^=0.09, *n*=75, *P*=0.01; Supplementary Fig. S1 at *JXB* online). Strong relationships were found for some genotypes (Flevo, *R*^2^=0.43, *n*=13, *P*=0.014; Lambro, *R*^2^=0.49, *n*=9, *P*=0.04; I214, *R*^2^=0.40, *n*=7, *P*=0.07), suggesting that, at the intra-genotype level, thicker roots grew faster than thinner ones. The first PCA plane highlighted the fact that all growth parameters covaried together and with ORER, especially EZ (Fig. 2B). Pairwise comparisons confirmed that the differences in ORER between poplar roots were better associated with the differences in EZ (*R*^2^=0.90, *P*<0.001; Fig. 2C) than with the differences in *P* (*R*^2^=0.77, *P*<0.001; Fig. 2D), in EER_max_ (*R*^2^=0.66, *P*<0.001; Fig. 2E), or in *N*_div_ (*R*^2^=0.62, *P*<0.001; Fig. 2F). Differences in EZ thus appeared to be a very good proxy for ORER variability.

### Variability in growth parameters across treated roots

In Exp2, the growth of Flevo roots was quantified in optimal nutrient solution (control) as well as in the presence of various chemicals (70 mM NaCl, 160 g l^−1^ PEG, 2 mM H_2_O_2_, 2 µM ABA or 10 µM ABA). The rationale was to assess whether chemically induced growth variations resulted either from the coordinated change of all growth parameters or from changes of only some of them. For each root, growth was documented at four successive time-points (within a 24-h period; Supplementary Fig. S2). The first time-point controlled for the inter-individual variability in root growth before treatment onset. Flevo growth rates tended to be lower but were less variable in Exp2 than in Exp1 (Exp2, ORER=0.70 ± 0.03 mm h^−1^, *n*=41, ranging from 0.39–1.06 mm h^−1^).

**Fig. 3.**
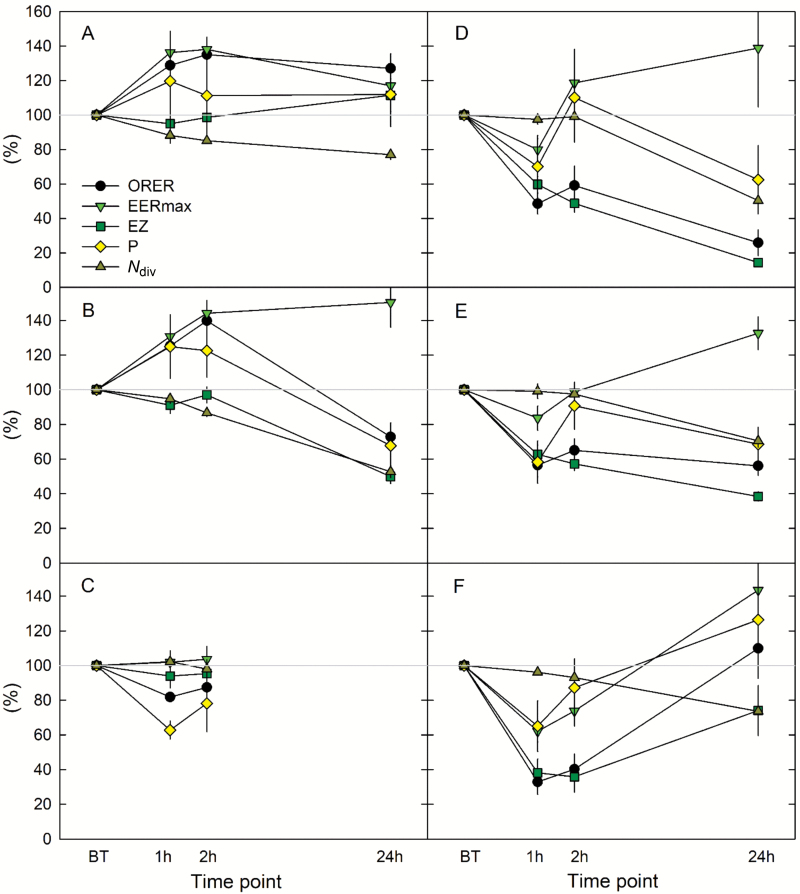
Response of root elongation rate and growth parameters to chemical treatments as a function of time (Exp2). Root growth was monitored at four successive time-points: before treatment onset (BT), and at 1, 2, and 24 h after treatment onset. Parameters are expressed as a percent of their initial values (i.e. at BT) to account for inter-individual variability. Data are means ±SE. Flevo roots were grown in optimal nutrient solution (control, *n*=5, A), supplemented with 2 µM ABA (*n*=12, B), 10 µM ABA (*n*=3, C), 70 mM, NaCl (*n*=8, D), 160 g l^−1^ PEG (*n*=5, E) and 2 mM H_2_O_2_ (*n*=8, F). Under 10 µM ABA, roots stopped growing before the last time-point.

We first addressed the question of the dynamics of growth response to each treatment (Fig. 3). Under control conditions, ORER increased during the 2 h following the cutting installation, and remained stable over successive time-points (Fig. 3A). EER_max_ and *P* showed similar kinetics while EZ and *N*_div_ remained close to their initial values. Applying 2 µM ABA did not modify the early dynamics of the growth parameters but strongly reduced ORER, *P*, and *N*_div_ after 24 h (Fig. 3B). At 10 µM, ABA treatment resulted in a more rapid response, which led to growth arrest at 24 h (Fig. 3C). These dynamics were consistent with a dose-dependent response that required time to become physiologically active. NaCl and PEG treatments induced similar dynamics in growth response, rapidly slowing root growth and reducing the EZ (Fig. 3D, E). A transient reduction in EER_max_ was observed 1 h after stress onset before a full recovery. *P* showed more complex dynamics over the successive time-points, being reduced at 1 h, recovering at 2 h, and again being reduced at 24 h. The responses to H_2_O_2_ were similar to those in the presence of PEG or NaCl at the earliest time-points, but most parameters recovered at 24 h (Fig. 3F). Such a two-phased response could reflect an acute response to an initial oxidative burst, followed by either acclimation or a release of oxidative stress.

**Fig. 4.**
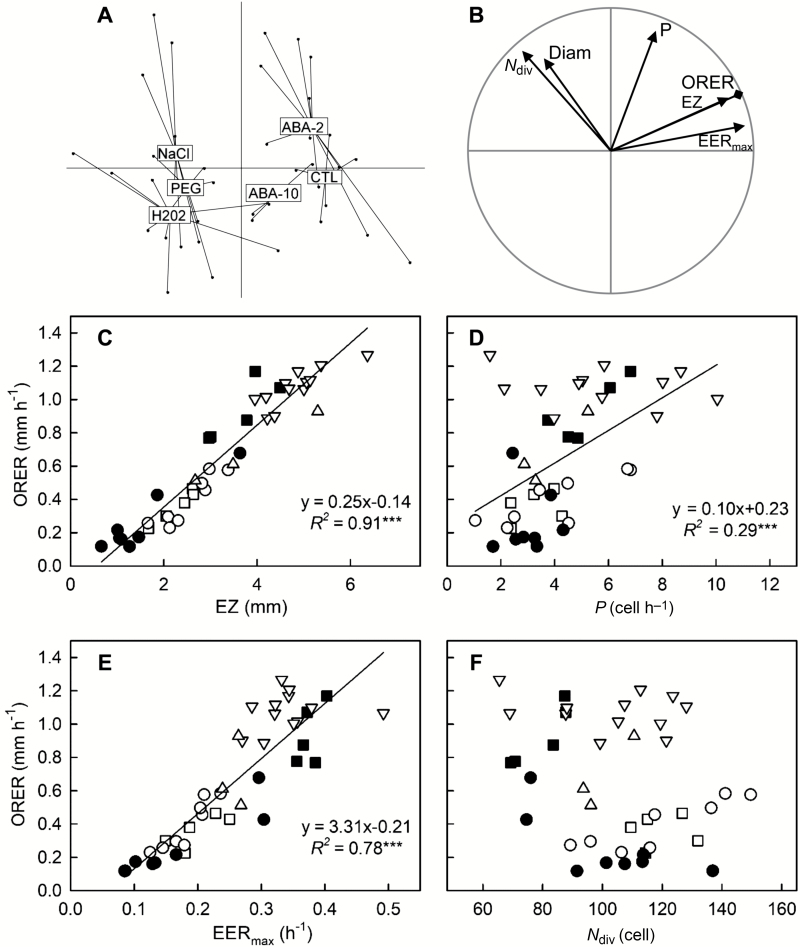
Covariation of growth parameters across chemically treated Flevo roots at the early time point. (A, B) Principal component analysis was performed on the growth parameters determined 2 h after treatment onset in Exp2. The first two main factors of the PCA account for 81% of the total variation. For clarity, individuals and variables are displayed on the same PCA plane on distinct figures. (A) Projection of the individuals in the F1xF2 plane. Each point is a root (*n*=41) and lines and boxes indicate the treatment barycentres. (B) Projection of growth parameters in the F1×F2 plane. *P*, cell production rate; *N*_div_, number of dividing cells; EZ, length of the elongation zone; EER_max_, maximal elemental elongation rate; and Diam: the apical root diameter. Overall root elongation rate (ORER) was added as a supplementary variable. (C–F) Pairwise relationships between ORER and growth parameters. The treatments are indicated as follows: closed squares, optimal nutrient solution (control); closed circles, 2 mM H_2_O_2_; open circles, 70 mM NaCl; open squares, 160 g l^−1^ PEG; inverted triangles, 2 µM ABA; triangles, 10 µM ABA. Linear regressions are plotted and the significance of the correlations is indicated (***P≤0.001).

**Fig. 5.**
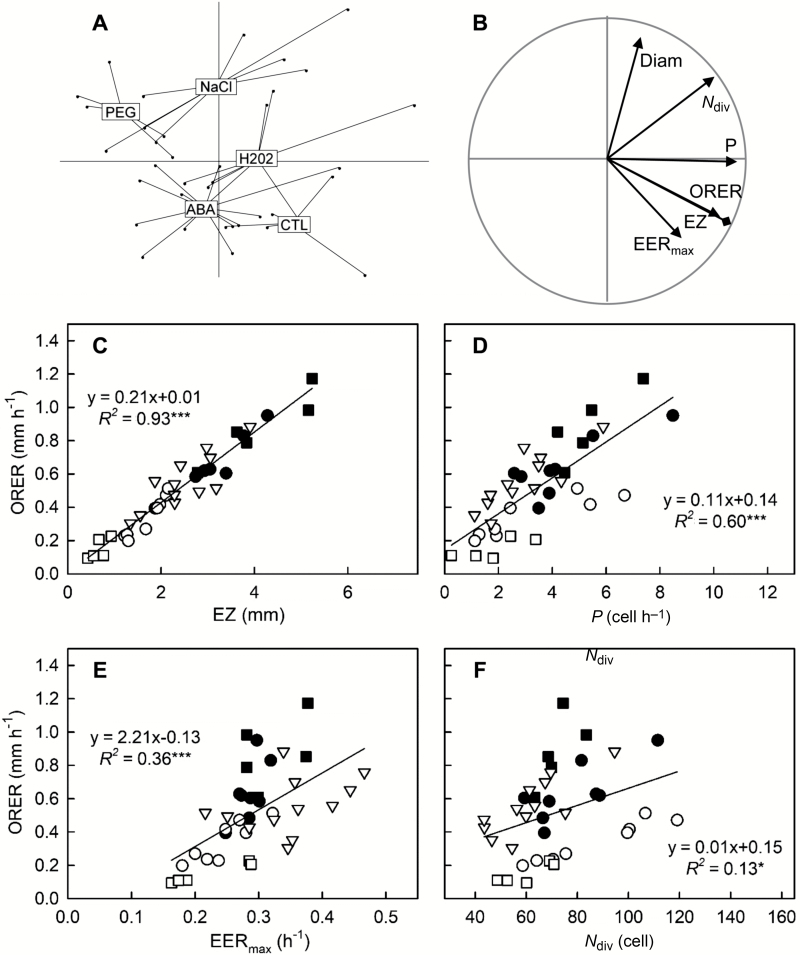
Covariation of growth parameters across chemically treated Flevo roots at the late time point. (A, B) Principal component analysis was performed on the growth parameters determined 24 h after the treatment onset in Exp2. The first two main factors of the PCA account for 79 % of the total inertia. For clarity, individuals and variables are displayed on the same PCA plane on distinct figures. (A) Projection of the individuals in the F1×F2 plane. Each point is a root (*n*=38). Lines and boxes indicate treatment barycentres. (B) Projection of growth parameters in the F1×F2 plane. *P*, cell production rate; *N*_div_, number of dividing cells; EZ, length of the elongation zone; EER_max_, maximal elemental elongation rate; and Diam, the apical root diameter. Root elongation rate (ORER) was added as a supplementary variable. (C–F) Pairwise relationships between ORER and growth parameters. Closed squares, optimal nutrient solution (control); closed circles, 2 mM H_2_O_2_; open circles, 70 mM NaCl; open squares, 160 g l^−1^ PEG; inverted triangles, 2 µM ABA. Linear regressions are plotted and the significance of the correlations is indicated (*P≤0.05, ***P≤0.001).

Due to their own dynamics and their differential impact on growth parameters, the chemicals increased the range of ORER among roots. Two hours after the onset of treatments, an 11-fold variation in ORER was found among roots, ranging from 0.12 mm h^−1^ for a H_2_O_2_-treated root to 1.27 mm h^−1^ for a 2 µM ABA-treated root. A 15-fold variation in ORER was found among roots 24 h after the onset of treatments, ranging from 1.17 mm h^−1^ for a control root to 0.10 mm h^−1^ for a PEG-treated root. To further assess how variation in individual growth parameters contributed to the variation in ORER, PCA was performed on the matrices of growth parameters and root diameter, using ORER as a supplementary variable (Figs 4, 5). Two time-points, 2 h and 24 h after the onset of treatments, were considered. At the earliest time-point (Fig. 4A, B), the first two PCA axes explained 81% of the total inertia, with PC3 accounting for 11%. PC1 was highly related to EER_max_ and EZ. The barycentres of treatments were gradually loaded along PC1, with no clear-cut discrimination on PC2, highlighting that the most structuring effect of treatment was due to variation in EER_max_ and EZ. Fast-growing roots (control and 2 µM ABA) were clearly separated on PC1 from slow-growing ones (H_2_O_2_, PEG, NaCl). ORER and EZ were superimposed (Fig. 4B), and EZ was again the best proxy for ORER (*R*^2^=0.91, *P*<0.001; Fig. 4C). In addition, ORER was better explained by EER_max_ (*R*^2^=0.78, *P*<0.001; Fig. 4E) than by *P* (*R*^2^=0.29, *P*<0.001; Fig. 4D). PC2 captured 36% of the total variance and was related mostly to *P* and partially to *N*_div_ (equally with PC1) and to Diam (equally with PC3). The loading of treatments on PC2 was not deeply structured, PC2 reflecting rather the constitutive inter-individual variability among roots. During the early response, ORER varied independently from Diam and from *N*_div_ (Fig. 4B, F).

The covariation pattern was modified drastically 24 h after the onset of treatments (Fig. 5). The data were structured mostly by variation in *P*, which was strongly and positively related to PC1. The first and second PC explained 48% and 31% of the total variance, respectively, with PC3 accounting for 14%. EZ and N_div_ contributed to PC1 and, to a lesser extent, to PC2. PC2 was related mostly to Diam (also contributing to PC3). EER_max_ contributed equitably to the three PC. Capturing 79% of the total inertia, the first two PCA axes clearly individualized the barycentres of the treatments (Fig. 5A). Similarly to the early response, ORER varied independently from Diam (Fig. 5B). ORER and EZ were superimposed (Fig. 5B), with EZ thus again being the best proxy for ORER (*R*^2^=0.93, *P*<0.001; Fig. 5C). Variation in ORER in response to the treatments was correlated to variation in *P* (*R*^2^=0.60, *P*<0.001; Fig. 5D) and, to a much lesser extent, with *N*_div_ (*R*^2^=0.13, *P*<0.05; Fig. 5F). Finally, ORER and EER_max_ were best captured on two distinct PC planes (Fig. 5F) and their covariation, while significant (*P*=0.36, *P*<0.001, Fig. 5E), was much less strong than during the early response.

### Cellular dissection of cell production rate variation

**Fig. 6.**
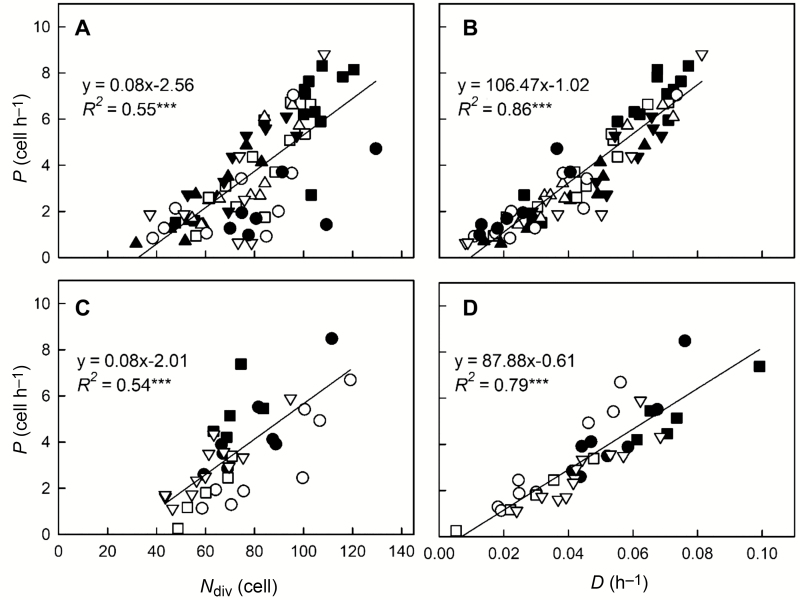
The cellular basis of differences in cell production rate across genotypes and chemically treated roots. (A, C) Relationships between the cell production rate (*P*) and the number of dividing cells (*N*_div_). (B, D) Relationships between cell production rate (*P*) and average cell division rate (*D*). For (A, B) the data are for different genotypes (*n*=75 roots, Exp1, see Fig. 2 for key to symbols). For (C, D) the data are for different treatments (*n*=38 roots, Exp2, 24 h after the onset of treatments, see Fig. 4 for key to symbols). Linear regressions are plotted and the significance of the correlations is indicated (***P≤0.001).

Our analyses showed that ORER variation in poplar root was related strongly to that of *P*, both across genotypes and across late responses to chemicals (Figs 2, 5). Assuming that all cells in the RAM are dividing, *P* depends on *N*_div_ and on cell division rate (*D*, h^−1^). Under steady state, *P* equals *N*_div_ times *D*. We tested which of these two growth parameters accounted for the 14- and 32-fold variations in *P* retrieved in Exp1 and Exp2 (24 h after treatment onset), respectively. While *N*_div_ explained about half of *P* variation (*R*^2^=0.55 and *R*^2^=0.54, *P*<0.001, respectively, in Exp1 and Exp2, Fig. 6A, C ), *D* explained more than 80% of *P* variation (*R*^2^=0.86 and *R*^2^=0.79, *P*<0.001, respectively, in Exp1 and Exp2, Fig. 6B, D). Together, *N*_div_ and *D* explained 99% of *P* variation (as expected) and the standardized coefficients suggested that *D* contributed more than *N*_div_ to *P* (beta=0.7 and 0.4, respectively, *P*<0.001).

## Discussion

In the present study, we quantified variation in the overall root elongation rate (ORER) across poplar genotypes and across chemically treated roots. We further determined several associated growth parameters, namely the length of the root apical meristem (RAM), the length of the elongation zone (EZ), the cell production rate by the meristem (*P*), the number of dividing cells (*N*_div_), the average cell division rate (*D*), and the maximal elemental elongation rate (EER_max_), and analysed their covariation patterns. High-resolution kinematics allowed the accurate and direct determination of some parameters such as the length of the zones or EER_max_ (Bizet *et al.*, 2015; Kopittke *et al.*, 2015; Royer *et al.*, 2016), whilst the calculated parameters *P* and *N*_div_ were determined from kinematics data and anatomical records, but were also directly estimated from analysis of an infrared reflectance profile and scaling factors (determined from typical cell lengths within the RAM). These proxies were weakly sensitive to the realistic range of values for the scaling factors. The high correlation between the parameters determined from raw data and their proxies showed that *P* and *N*_div_ could be estimated using a non-invasive method that required very low experimental effort (Fig. 1). In addition to saving time, this also allowed the monitoring over time of parameters that usually require destructive measurements. These proxies can be used in future studies and in other species assuming (or testing) that the same scaling factors are valid and that the treatments do not affect cell size in and at the boundary of the RAM.

Growth was studied both under steady-state conditions (genotype comparison, stabilized growth 24 h after the onset of treatments) and during a physiological response (1–2 h after the onset of treatments). The determination of ORER, EZ, and EER_max_ does not require time-invariance: these parameters reflect growth between two time-points and the time lapse only is critical for the proper assessment of EER along the root apex (Silk, 1984). Under non-steady situations, the computation of *P*, *N*_div_, and *D* requires to assess the rate of cell density change (Silk, 1984, Fiorani and Beemster, 2006). The magnitude of cell density change was null in response to PEG (Bizet *et al.*, 2015) and negligible in response to cold (Yang *et al.*, 2017). Here, the distribution of meristematic cell length was hardly modified 24h after treatments onset. The change of cell density within the RAM was thus assumed to be small enough during the short-term responses to be neglected. Our approach, based on high-resolution kinematics and proxies, allows monitoring of all growth parameters insofar as the rate of cell density change within the RAM is negligible.

The analyses of correlations between ORER and growth parameters highlighted that *P* was a stronger driver of variations in ORER among individual poplar roots than EER_max_ (Figs 2, 5). Similarly, Gázquez and Beemster (2017) found that differences in root elongation rate among six species were driven primarily by variation in *P*. In a previous study, *P* and cell elongation (through mature cell length) were found to contribute similarly to the variation of root elongation rate in 18 accessions of Arabidopsis (Beemster *et al.*, 2002). Moreover, the genetic variation of *P* was determined by variation in cell cycle duration and, to a lesser extent, by differences in *N*_div_. This is also in agreement with our data, with *P* being better correlated to the average cell division rate (*D*) than to *N*_div_ across both the roots of the eight genotypes and roots under chemical treatments (Fig. 6). In a recent meta-analysis of annual and biannual species, it appeared that the difference in *P* was determined equally by *N*_div_ and *D* (Gázquez and Beemster, 2017). Thus, in roots, *D* appears to be a key determinant of *P* and, consequently, of ORER.

While the elemental elongation rate varies along the root apex, showing a bell-shaped curve (Silk and Bogeat-Triboulot, 2014), EER_max_ was a good descriptor of the elongation capacity. EER_max_ varied from 10–40% h^−1^ among the different roots and was positively correlated to ORER, although less than *P* (Figs 2, 5). It became highly correlated to ORER during the short-term growth responses to chemicals, 2 h after treatment onset (Fig. 4). EER responded far more rapidly and thus drove root elongation rate more than cell production during the transition phase. Although independent from growth parameters, root diameter was slightly correlated with ORER when considering all genotypes, and positive correlations were found for two genotypes (Supplementary Fig. S1). The range of ORER for fine roots was lower than that of thick roots. As found in oak by Pagès (1995), the root apical diameter provided an upper limit to ORER and reflects more a potential than actual growth rate (Pagès *et al.*, 2010). Finally, there was no significant difference of ORER variability among the genotypes, and *P. nigra* and *P alba* genotypes had similar ORERs to commercial hybrids (except for one, Flevo). Poplar hybrids are selected mainly for high aerial productivity, disease resistance, and less so for rooting capacity (Zalesny and Zalesny, 2009). Our results suggest that the hybrid vigour of the shoot does not correspond with a higher growth rate of adventitious roots.

The root elongation rate was rapidly inhibited by oxidative, salt, and osmotic stress as well as by the addition of 10 µM ABA (Figs 3, 4). The addition of 2 µM ABA affected neither the growth parameters nor the ORER within the first hours as compared to controls. In line with this, H_2_O_2_ supplied in excess reduced root growth in tomato and Arabidopsis (Dunand *et al.*, 2007; Ivanchenko *et al.*, 2013), and osmotic and salt stress also decreased root growth (West *et al.*, 2004; Geng *et al.*, 2013; Shelden *et al.*, 2013; Royer *et al.*, 2016). In Arabidopsis and rice, application of ABA at a low concentration (0.1 µM) stimulated root growth, while 10 µM ABA strongly reduced it (Xu *et al.*, 2013). A similar dose-response to ABA has been reported in maize (Mulkey *et al.*, 1983). Here, at the early time-point, the variation in ORER across chemically treated roots was driven more by the variation in EER_max_ than by that in *P* (Fig. 4). Rapid growth adjustment in response to exogenous cues was related more to EER than to cell proliferation, although both contributed (Fig. 4). According to a 1-h-long monitoring run 24 h after the onset of treatment, the root elongation rates of all treatments returned to a steady state (data not shown). The full recovery of all parameters under H_2_O_2_ treatment could be due to fading of this chemical in the nutrient medium after this time (Ivanchenko *et al.*, 2013). The growth rate of roots under osmotic and salt stress remained reduced and was finally affected under ABA treatment. At that time, ORER was correlated strongly with *P* and, to a lesser extent, with EER_max_ (Figs 3, 5), a situation close to the comparison of ORERs across genotypes (Fig. 2). The relative contribution of *P* to ORER over the kinetics of responses to the treatments could be related to the differential responsiveness of *D* and *N*_div_ over time. The early variations of *P* in response to stress have been found to be due mainly to variations in *D*, with *N*_div_ requiring a longer time to change (West *et al.*, 2004; Bizet *et al.*, 2015). At later stages, variations in *P* were due to the RAM shortening while *D* had fully recovered (West *et al.*, 2004; Liu *et al.*, 2013). It has even been suggested that the rapid reduction of *D* is involved in the later reduction of *N*_div_ (Gázquez and Beemster, 2017).

From the point of view of kinematics, ORER is the area below the EER profile along the growth zone, and EER is low in the RAM (Bizet *et al.*, 2015). ORER is thus proportional to the product of EER_max_ and EZ. In the early response to metal toxicity, the alteration of ORER was due to a reduction of EER_max_ and, to a lesser extent, of EZ (Kopittke *et al.*, 2015; Kopittke and Wang 2017). Here, EER_max_ contributed to ORER, but ORER was incredibly well correlated with EZ, regardless of the experiment, whether it was a genotype comparison or the early or late responses to external cues (Figs 2, 4, 5). In a review, Baskin (2013) noted that there are more examples in the literature of regulation of ORER through variation of EZ than through variation of EER, and suggested that changes in EER could be an indirect consequence of an active change in the location of elongation ending. While the spatial control of transition from proliferation to elongation under the influence of hormonal interplay is now well understood, little is known about the control of where elongation ends (De Vos *et al.*, 2014; Gázquez and Beemster, 2017). The Control of elongation ending through GA dilution in the expanding cells has been proposed as a cell-autonomous model (Band *et al.*, 2012) but other models support the involvement of spatial control (De Vos *et al.*, 2014). Meanwhile, the duration of rapid cell elongation (the time taken for a cell to cross the EZ) was not affected in response to osmotic and salt stress (Sharp *et al.*, 1988; West *et al.*, 2004). Under a constant duration of cell elongation, the reduction of EZ could simply result from the reduced *P* and EER, i.e. there will be fewer expanding cells in the elongation zone and the lower relative elongation rate will reduce their size. Indeed, EZ comprises (1) the number of expanding cells, which depends directly on the functioning of the RAM that feeds the elongation zone, (2) expansion duration, which is translated into length by kinematic action, and (3) EER, which affects individual cell length within the elongation zone. The variations in EZ explain most of the variations in ORER. EZ can thus be regarded as the most synthetic parameter, integrating the activity levels of both processes into a single parameter.

In conclusion, the use of proxies allowed the rapid and non-destructive quantification of meristem activity. High-quality imaging enabled the cell production rate to be computed from experimental data obtained directly at the shootward border of the RAM. One outcome of our work is that the functioning of the root meristem can now be quantified continuously over time and even in response to fluctuating environments. While cell production rate was found to be the main driver of growth rate, the elemental elongation rate was a key driver of short-term growth adjustment. The length of the elongation zone, integrating the activity of both processes, was a proxy for root elongation rate.

## Supplementary data

Supplementary data are available at *JXB* online.

Fig. S1. Relationships between root apical diameter and overall root elongation rate across genotypes (Exp1).

Fig. S2. Time-course of the root elongation rate response to chemical treatments (Exp2).

Supplementary Figures S1-S2

## Abbreviations:

ABA: abscissic acid
CPR: local rate of cell production
Diam: root apical diameter
D: average cell division rate
EER: elemental elongation rate
EZ: length of the elongation zone
H_2_O_2_: hydrogen peroxide
*N*_div'_: number of dividing cells
P: cell production rate
RAM: root apical meristem
ORER: overall root elongation rate
