# Supplementary material for: Quantitative dissection of variations in root growth rate: a matter of cell proliferation or of cell expansion?
Source: J Exp Bot. 2018 Jul 25;69(21):5157–68. doi: 10.1093/jxb/ery272 (PMC6184812; doi:10.1093/jxb/ery272)
Supplement: Supplementary Figures S1-S2 [file ery272_suppl_supplementary_figures-s1-s2.pdf]

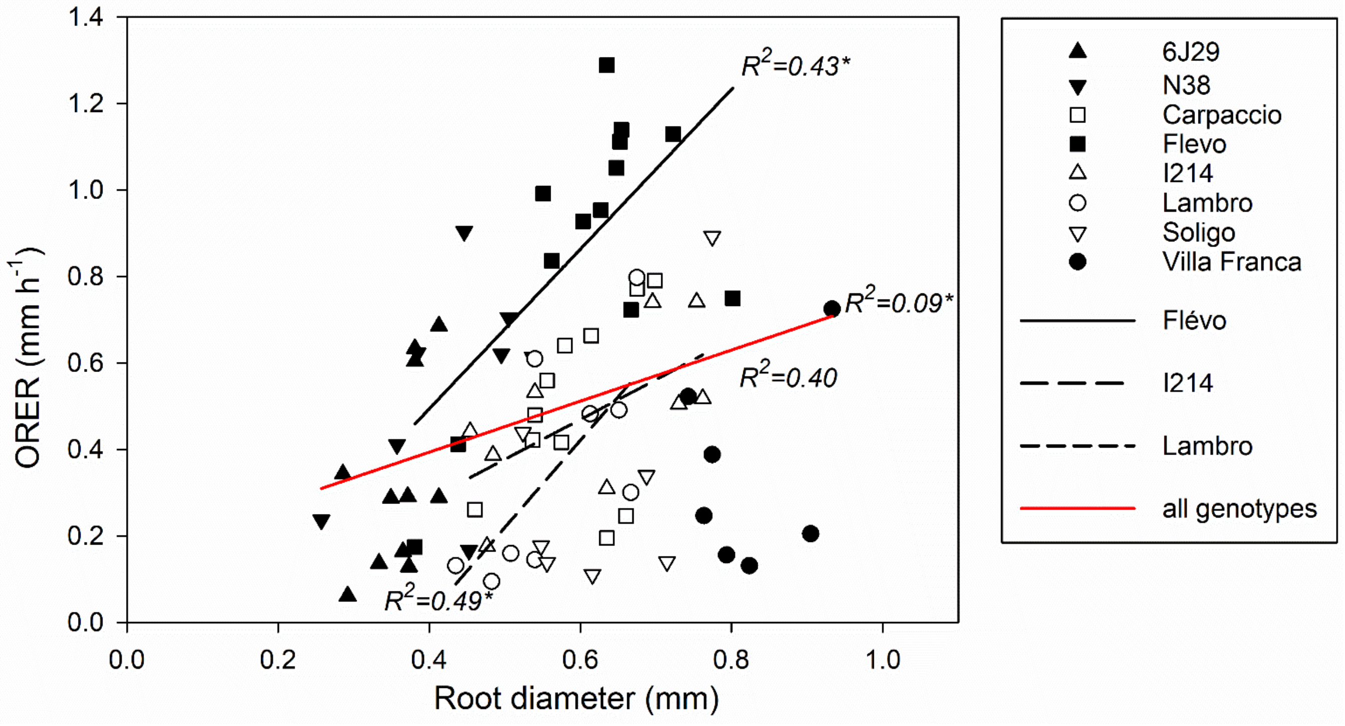

Figure S1. Relationship between root apical diameter and overall root elongation rate (ORER) across genotypes (Exp1).

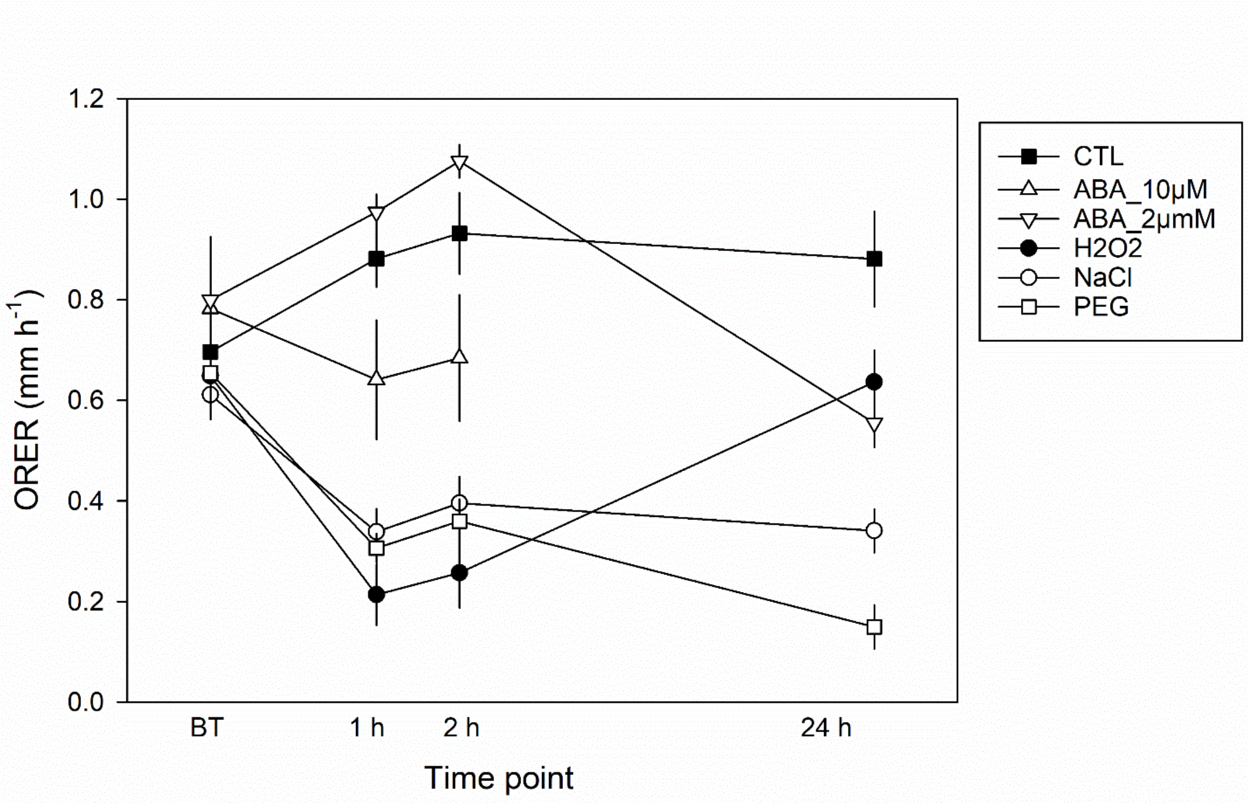

Figure S2. Time-course of root elongation rate response to chemicals (Exp2). Root growth was monitored at four successive time points: before treatment onset (BT) and 1 h, 2 h and 24 h after treatment onset. Data are mean ± SE. Flevo roots were grown in optimal nutrient solution (CTL, n = 5), supplemented with 2 mM H_2O_2 (n = 8), 70 mM NaCl (n = 8), 160 g l^{-1} PEG (n = 5), 2 µM ABA (n = 12) and 10 µM ABA (n = 3). Under 10 µM ABA, roots stopped growing before the last time point.
